# Adult Onset Hypertrophic Cardiomyopathy (HCM) Not Detected by Echocardiogram: A Case Presentation

**DOI:** 10.7759/cureus.45932

**Published:** 2023-09-25

**Authors:** Kawther N Elsouri, Jerry Camacho Ramos, Kevin Stepanek, Aydin Turan, Marc M Kesselman, Michelle L Demory

**Affiliations:** 1 Osteopathic Medicine, Nova Southeastern University Dr. Kiran C. Patel College of Osteopathic Medicine, Fort Lauderdale, USA; 2 General Practice, Nova Southeastern University Dr. Kiran C. Patel College of Allopathic Medicine, Fort Lauderdale, USA; 3 Internal Medicine, Trinity Health Oakland Hospital, Pontiac, USA; 4 Rheumatology, Nova Southeastern University Dr. Kiran C. Patel College of Osteopathic Medicine, Fort Lauderdale, USA; 5 Microbiology and Immunology, Nova Southeastern University Dr. Kiran C. Patel College of Allopathic Medicine, Fort Lauderdale, USA

**Keywords:** adult onset hcm, diastolic dysfunction, cardiology imaging, transthoracic echocardiogram, hypertrophic obstructive cardiomyopathy (hocm)

## Abstract

Hypertrophic cardiomyopathy (HCM) is a genetic myocardial disease of the sarcomere protein. The age of diagnosis of HCM tends to be between the second to third decades of life. However, the recent occurrence of HCM in the fifth and sixth decades of life has been seen in an increasing number of cases. In all cases, a transthoracic echocardiogram (TTE) is considered the gold standard of imaging. Here, we present a case of a 54-year-old Caucasian male who presented to the emergency department (ED) with dyspnea while on vacation. An electrocardiogram (ECG) taken at the time did not suggest any abnormalities. After returning home, a stress test conducted indicated left anterior descending (LAD) artery stenosis. Following treatment, symptoms improved temporarily but eventually came back. Repeat ECGs and TTEs done over the next two years indicated grade II diastolic dysfunction and mild left ventricular hypertrophy, which led to changes in the medication regime. Nevertheless, his condition progressively deteriorated over time. Repeat appearances to the ED led to the utilization of magnetic resonance imaging (MRI) to assess cardiac morphology function and velocity flow. The results were consistent with HCM. This case presents a unique obstacle for the diagnosis of adult-onset HCM. The change made to his medication regimen seemingly aggravated the patients’ condition. This case highlights the need for further imaging, beyond the gold standard, in adult males with repeated complaints of dyspnea on exertion (DOE).

## Introduction

Hypertrophic cardiomyopathy (HCM) or hypertrophic obstructive cardiomyopathy (HOCM) is a genetic myocardial disease involving the sarcomere protein [1]. Mutations in the sarcomere protein can involve genes encoding for cardiac myosin binding protein-C, cardiac troponin I, or α-cardiac myosin heavy chain [1]. Dysfunction of the sarcomere protein, which is responsible for the basis of the structural integrity of skeletal/cardiac muscle, leads to cardiac hypertrophy and left ventricular obstruction [1]. The majority of cases follow an autosomal dominant inheritance pattern [1]. Certain genes have been identified to be associated with an increased risk of developing HCM [2]. However, each gene’s variable penetrance poses a challenge in fully understanding the underlying complexities regarding the nature of this condition [2].

The diagnosis of HCM is confirmed with the presence of a left ventricular wall thickness of ≥15 mm that is otherwise unexplained by abnormal loading conditions (e.g., hypertension, valvular, and congenital disease) [1]. Common symptoms of HCM can include dyspnea, chest pain, palpitations, and syncope with risks, including sudden cardiac death, heart failure, and atrial fibrillation [1]. Oftentimes, patients with HCM present in various ways. Some remain asymptomatic or mildly symptomatic and present due to family history, detection of a murmur, or an abnormal electrocardiogram (ECG).

Some patients develop an abnormal diastolic function, and complications can include increased left ventricular pressures that impair ventricular filling, which may further exacerbate obstruction [3]. Diastolic dysfunction found in HCM is primarily due to myocardial hypertrophy and fibrosis [4]. This, in combination with outflow tract obstruction and ventricular stiffness, results in an increased risk for myocardial ischemia [4]. Typically, the age of diagnosis of HCM is between the second to third decades of life [5]. Although the occurrence of HCM in the fifth and sixth decades of life has traditionally been considered rare, there have been an increasing number of cases of late age diagnosis recently [5]. In all age groups, transthoracic echocardiogram (TTE) has an 80% accuracy for the diagnosis of HCM and is considered a gold standard and diagnostic for this disease, while being erroneous for the remaining 20% [3]. This gap in sensitivity can be fatal in such a disease. As diagnostic and therapeutic regimes evolve, clinicians will need to become familiar with the various imaging modalities (other than TTE) and how they can be useful when assessing presentations of this condition. In this report, we present a case of a 54-year-old male with a unique presentation of HCM, which was not initially indicated by his TTE. This case highlights the utility of other diagnostic tools outside of those traditionally accepted for HCM and discusses possible factors possibly influencing the onset of this case.

## Case presentation

**Figure 1:**
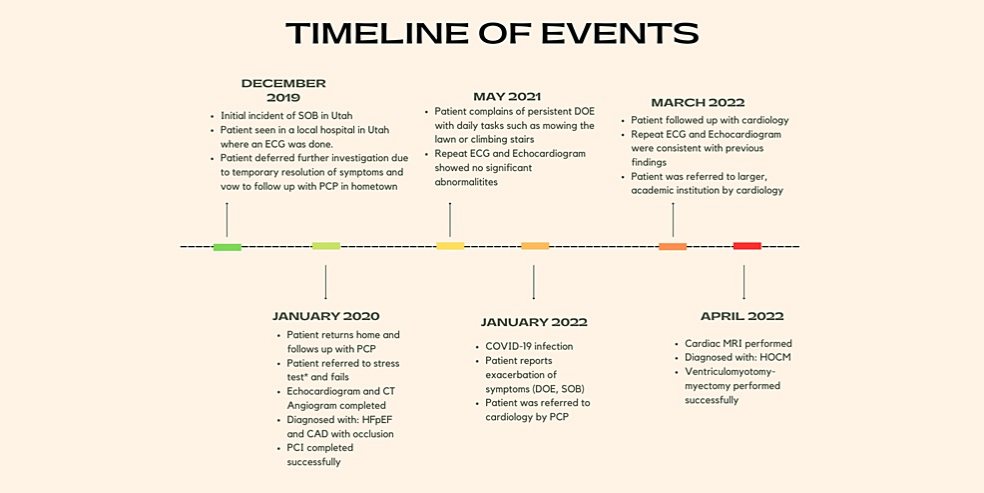
Timeline of events leading to diagnosis and surgical intervention. ECG: 12-lead electrocardiogram; DOE: dyspnea on exertion; SOB: shortness of breath; PCP: primary care provider; CT: computerized tomography; HFpEF: heart failure with preserved ejection fraction; CAD: coronary artery disease; cardiac MRI: cardiac magnetic resonance imaging; HOCM: hypertrophic obstructive cardiomyopathy Original image created with canva.com.

A 54-year-old Caucasian active male with past medical history of irritable bowel syndrome, hyperlipidemia, coronary artery disease, hypertension, obstructive sleep apnea (OSA), tobacco use disorder, and restless leg syndrome presented to his primary care provider (PCP) for a routine annual physical exam in December 2019. The patient did not report any family history of HCM or congenital heart defects. No audible murmurs, palpable thrills, or peripheral edema were noted in the physical exam. At the time, the patient denied headaches, dizziness, palpitations, and chest pain. In addition, an ECG was performed, and results indicated a regular rate and sinus rhythm. Two weeks later (Figure 1), when on a ski vacation in Salt Lake City, Utah, he experienced severe dyspnea and was evaluated at a local hospital emergency department.

The patient’s initial workup included an ECG, which was negative for ST elevation and bundle branch blocks and demonstrated a normal sinus rhythm with nonspecific changes. Due to personal choice, the patient decided to continue the workup for this event in his home state with his PCP. After returning home from the Utah vacation, a thallium nuclear exercise stress test was ordered, which resulted in a recurrence of symptoms (dyspnea and central chest pressure). For this reason, the test was aborted after seven minutes and 26 seconds (maximum mets = 9.2). The results indicated reversible ischemia in the apical inferior aspect of the left ventricle with a normal ejection fraction. He was sent directly to the emergency department for evaluation and cardiac consultation. In the ER, IV heparin was initiated in preparation for angiographic imaging. Doppler TTE was performed at bedside along with right and left heart catheterization. While imaging was unable to be retrieved for visualization in this report, the interpreted results of both modalities were consistent with the diagnosis of acute heart failure with preserved ejection fraction (HFpEF). In addition, the angiographic findings included coronary artery disease with 75% stenosis of the proximal left anterior descending (LAD) artery. Left ventricular end diastolic pressure (LVEDP) was elevated (LVEDP = 27 mmHg). However, the ejection fraction was preserved (EF= 65%). Mitral valve structure was noted to be normal with trace regurgitation. The aortic valve was observed as a tri-leaflet that exhibited normal excursion without thickening, regurgitation, or stenosis. In addition, no wall motion abnormalities were detected. Percutaneous catheter intervention (PCI) was undertaken, and a drug-eluting stent was placed in the proximal LAD artery. Stenosis was reduced to 0%. At the time, the treatment regimen included atorvastatin 40 mg, metoprolol XL 25 mg, fenofibrate 160 mg, spironolactone 25 mg daily, lisinopril 40 mg daily, nitroglycerin 0.4 mg taken as needed, furosemide 40 mg, and diltiazem 180 mg daily.

**Figure 2:**
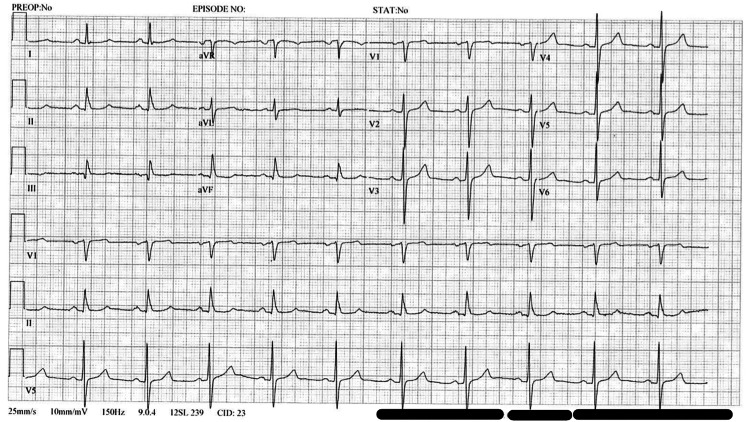
Electrocardiogram (ECG) from May 2021 Electrocardiogram (ECG) demonstrating a sinus rhythm at a rate of 64 BPM, small Q waves on leads II and III and aVF, and increased voltage (suggestive of left ventricular hypertrophy). Imaging obtained from centralized electronic medical records with the consent of the patient.

**Figure 3:**
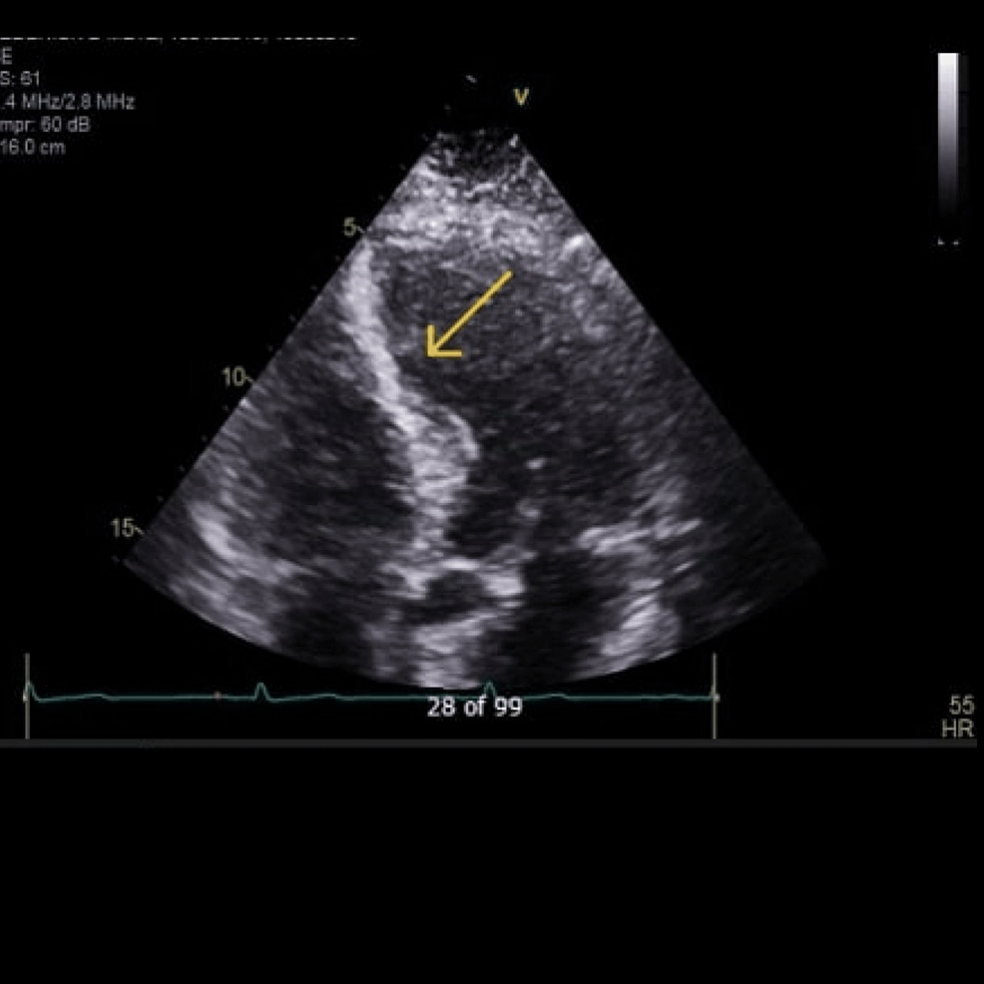
Echocardiogram from May 2021. Yellow arrow pointing to the interventricular septum. Transthoracic echocardiogram (TTE) complete with contrast, bubble, strain, and 3D PRN order panel (May 2021). Acquisition window and orientation are an apical four-chamber view, utilizing Change Healthcare Cardiology Software (CPACS) as the imaging tool/imaging mode. Left ventricle (LV) cavity size is normal with mild concentric hypertrophy. There is grade I (mild) diastolic dysfunction and normal left atrial pressure. Visually estimated ejection fraction is 65%. There are no LV wall motion abnormalities noted. LV mass and mass index (2D) are 179 g and 77 g/m^2^, respectively. Left ventricle wall thickness is noted as normal. LV outflow tract (LVOT) stroke volume and LVOT stroke index are 139 g and 60 mL/m^2^, respectively. No significant changes were observed when compared to previous TTE imaging. Imaging obtained from centralized electronic medical records with the consent of the patient.

Initially, the patient reported a mild improvement in symptoms. However, by May 2021, he continued experiencing difficulty completing daily tasks, such as mowing the lawn or climbing stairs without dyspnea. A repeat ECG (Figure 2) was performed, which found no significant changes and indicated normal sinus rhythm. A concurrent TTE did not show significant abnormalities contributing to presenting symptoms (Figure 3).

**Figure 4:**
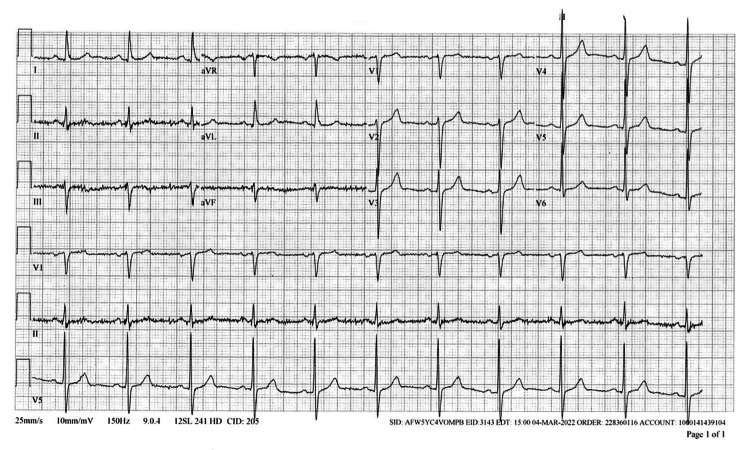
ECG from March 2022. Electrocardiogram (ECG) demonstrating sinus rhythm at a rate of 65 BPM, small Q waves on leads I and II, aVL, aVF, and increased voltage (suggestive of left ventricular hypertrophy). Baseline interference identified in leads II and III and aVF. Imaging obtained from centralized electronic medical records with the consent of the patient.

**Figure 5:**
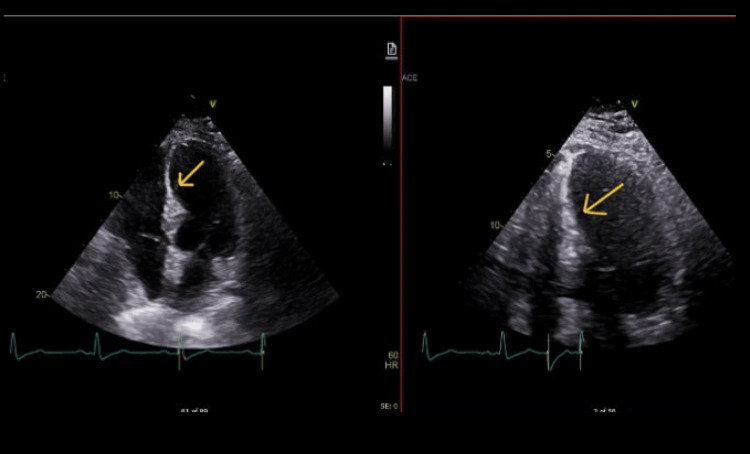
Echocardiogram from March 2022. Yellow arrows pointing to the interventricular septum. Transthoracic echocardiogram (TTE) complete with contrast, bubble, strain, and 3D PRN order panel (March 2022). Acquisition window and orientation are an apical five-chamber view, utilizing the Change Healthcare Cardiology Software (CPACS) as the imaging tool/imaging mode. Left ventricle (LV) cavity size is normal. The visually estimated ejection fraction is 70%. There are no regional LV wall motion abnormalities noted. LV mass and mass index (2D) are 98 g and 42 g/m^2^, respectively. The LV wall thickness is noted as normal. The LV outflow tract (LVOT) stroke volume and LVOT stroke index are 136 g and 59 mL/m^2^, respectively. No significant changes were observed compared to previous imaging. Imaging obtained from centralized electronic medical records with the consent of the patient.

In January 2022, the patient’s PCP diagnosed him with SARS-CoV-2 infection. The patient was not vaccinated and received a five-day course of nirmatrelvir/ritonavir and prednisone. The patient did not report any adverse effects from the treatment. However, he noted that his cardiac symptoms worsened following recovery of SARS-CoV-2. By March 2022, he presented to his outpatient cardiologist for follow-up. At that time, he presented with progressive lightheadedness, chest pain, and dyspnea on exertion (DOE). An ECG and TTE were performed. The ECG (Figure 4) results indicated a sinus rhythm at a rate of 65 BPM, small Q waves on leads I and II, aVL, aVF, and increased voltage (suggestive of left ventricular hypertrophy). Baseline interference was identified in leads II and III and aVF. No significant abnormalities were noted on TTE (Figure 5). His medication regime remained the same.

Due to the patient’s reported chronic symptoms and lack of access to proper imaging modalities, he was referred to a larger, academic institution. Within three weeks, he followed up and reported dyspnea on relatively minimal exertion, which was worsening over time, as well as intermittent left-sided heaviness in his chest. The heaviness was described as radiating to the shoulder and lasting for hours. These symptoms had progressively climaxed in severity since meeting with his home state cardiologist weeks previously. The patient noted that over time, the frequency of use of his oral nitroglycerin (to help alleviate chest discomfort) had increased. Consistent with previous findings, a repeat TTE revealed the impression of Grade II left ventricular diastolic dysfunction with a hyperdynamic left ventricular systolic function. Moderate concentric left ventricular hypertrophy was noted with a normal function and size of the right ventricle. The pulmonary venous pattern showed a blunted systolic flow, and trace mitral and tricuspid valve regurgitation were noted. Due to the history of DOE, further workup was utilized to quantitate left ventricular and valvular function.

A Philips Achieva® 1.5 Tesla magnetic resonance imaging (MRI) scanner was utilized for the cardiac morphology function and velocity flow. Findings showed moderate to severe asymmetric septal hypertrophy (also known as ASH, a previously known subtype of HCM) measuring up to 1.9 cm in the basal septum consistent with HOCM. The results showed no segmental wall motion abnormalities of the left ventricle. Quantitative left ventricular functional values (end diastolic volume (EDV), end systolic volume (ESV), stroke volume, left ventricular ejection fraction (LVEF), cardiac output, cardiac index, and left ventricular mass) were all within the range for normal limits. Subtle “sand-like” mid-myocardial spots were noted in the thickened septum, which, although non-specific, may appear in HCM. Otherwise, there was no evidence of prior myocardial damage due to ischemia. The aortic valve was unrestricted, and the mitral valve appeared structurally normal (with mild to moderate regurgitation directed posteriorly). There was systolic anterior motion of the mitral valve, with near mitral septal contact resulting in a significant flow acceleration within the left ventricular outflow tract. In addition, there were findings of mild ectasia of the mid-ascending thoracic aorta (4.0 cm). The right ventricle size and systolic function were noted to be normal.

Furosemide was discontinued, as it was seemingly associated with extreme fatigue and exacerbation of symptoms. After the diagnosis, ventriculomyotomy-myectomy was performed, and up to eight grams of septal tissue and an incidental growth was removed and sent for biopsy. Pathology of the same noted that the sample taken was non-malignant and was perceived as an incidental finding. Additional findings during surgery were consistent with HCM. The results of the procedure, in addition to symptoms and imaging, allowed for the definitive diagnosis of HCM over other diagnoses. As many mutations can be associated with HCM, further genetic investigation was offered. However, the patient opted to not proceed with genetic testing. The patient's self-reported symptoms improved rapidly within one month post-operation, and progress is currently being monitored by his PCP.

## Discussion

HCM is the most common cause of sudden death in the young and has disproportionately been excluded from possible diagnosis in those past the age of 40 [5]. However, recent literature has discovered that this phenomenon is not limited to a specific age range. This shift in paradigm has been thought to be due in part to increased clinical suspicion in conjunction with advanced cardiac imaging [6]. 

HCM is a genetic disease with the possibility of fatal outcomes. Genetic mutations lead to increased production of cardiac muscle, resulting in LV hypertrophy and LV outlet tract obstruction [4]. HCM primarily exhibits an autosomal dominant inheritance pattern [1]. Among the known genetic variants involved with HCM, there are eight mutations that are considered validated without controversy [2,7]. However, there is a wide array of mutations that have not achieved this status, for their complex underlying mechanisms are yet to be understood [2]. Once this milestone is achieved, it may open the door for exploring genetic approaches for treating HCM. Such therapies include exogenous gene replacement, RNA interference, and gene editing techniques, such as CRISPR-Cas9 [8]. 

Regardless of the age of onset, there exists a persistent misunderstanding that HCM is not only grim and unrelenting but increasingly progressive throughout life due to such mutations [6]. Recent data seem to change perceptions of the natural course of HCM [6]. HCM-related death/event rates owing to progressive heart failure, embolic stroke, or sudden death in patients of more advanced age (>60 years) are less than rates reported at other ages, and the estimated annual mortality risk is less than that in the general population matched for age and sex [9,10]. The natural course of HCM demonstrates stability in this advanced age group with almost 80% of surviving patients reporting no or mild HCM-related heart failure symptoms [6]. 

Anecdotally, TTE is regarded as the gold standard for the diagnosis of HCM [11]. However, more recent research demonstrates many shortfalls of TTE in identifying various patterns of left ventricular hypertrophy [12]. Particularly, TTE underestimates the magnitude of hypertrophy in various regions of the heart (anterolateral) and the presence of extreme LV wall thickness (≥30 mm) [12]. Currently, cardiovascular MRI (cardiac MRI) has been employed by many as the suggested alternative for improved diagnostic accuracy [13,14]. A study comparing these two imaging modalities in 103 HCM patients has found that cardiac MRI possessed a higher accuracy than in detecting abnormalities in the interventricular septum [15]. It was found that cMRI specificity and sensitivity in identifying abnormal muscle bundles was 97.1% and 100%, respectively [15]. By contrast, TTE demonstrated a sensitivity and specificity of 36.9% and 95%, respectively [15]. Moreover, this study highlights the underestimation of TTE in the maximum average Interventricular septum thickness by up to 7.3 cm (standard deviation (SD) 4.8) [15]. These differences in performance should warrant a re-evaluation in determining the gold standard. The unique expression and erratic onset of symptoms reported in this case set multiple boundaries. While HCM has been found to be more common later in life, its prevalence ranges between 0.16% and 0.29% of the general population [16]. In addition, the current case does not demonstrate any abnormal imaging or signs/symptoms prior to 2019 indicative of any obstructive cardiomyopathy. Given this information, patient demographics, and the diastolic dysfunction (commonly occurring in HCM [17]), diagnosis seemingly pointed to diastolic heart failure. Typically, diastolic heart failure is treated by decreasing fluid overload, thereby relieving associated symptoms (e.g., DOE, edema, and hypertension) [18]. However, in this patient’s case, the disease worsened significantly. The decrease in volume due to furosemide in HCM can lead to a decrease in stroke volume, worsening the left ventricle outflow tract gradient, which causes lightheadedness, dizziness, and syncope. Ironically, rather than triggering an arrhythmia, which could have led to sudden cardiac death (the most common cause of death in HCM) [18], the use of furosemide contributed to the discovery and ultimate resolution of the underlying condition. As diastolic heart failure can be diverse in its etiology, factors including left and right ventricular relaxation, chamber stiffness and impaired relaxation leading to a filling impairment, and elevated atrial filling pressures can pose a conundrum to pharmacological treatment.

That being said, other findings should be considered. A benign growth (also referred to as myxoma in this article) was noted during the septal myectomy. The patient had been aware of a cardiac growth for many years previous to 2019 with the understanding of its incidental nature and minor impact on his long-term health. While there was no clear explanation as to the histology of this growth in comparison to septal tissue, biopsy results confirmed non-malignant pathology. A previously reported case highlights the onset of a cardiac myxoma in a patient with COVID-19 [19]. Considering that the patient’s signs and symptoms began to worsen following a COVID-19 infection, it is possible that COVID-19 in addition to the existing myxoma possibly contributed to disease progression and onset. Many myxomas will not cause symptoms and are often discovered when an imaging study is done for another reason [19]. Regardless of etiology, the removal of said myxoma was necessary to decrease acute and chronic risk of disease complications. While most myxomas are benign, considering the presenting symptoms, the septal enlargement should have been further investigated. Future research should aim to identify possible triggers, such as infections, associated with the later onset of HCM in older populations.

## Conclusions

Our patient’s age and comorbidities, in addition to imaging, repeatedly signaled diastolic heart failure. After being treated for diastolic heart failure, symptoms did not improve, prompting further workup. Diagnosis in this case was dependent upon imaging results from cardiac MRI. While this modality has proven to be superior in the established literature, many providers and hospitals have limited access to such advanced imaging. The patient subsequently underwent surgical removal of his hypertrophic interventricular septum muscle. Only then did his symptoms significantly remit and his quality of life improved. This case elucidates the danger of repeatedly utilizing common imaging modalities in complex cases, such as this one. In addition, our case demonstrated the importance of taking multiple variables into consideration when analyzing for the onset and worsening of HCM in the adult population. Those discussed were the role of genetics, COVID-19, and benign cardiac growth in disease manifestation.

It is possible that the misinterpretation and redundancy of repeat imaging prolonged accurate diagnosis. It is equally likely that the traditional TTE employed in this case, due to their two-dimensionality, did not sufficiently portray hypertrophy in order to provide sufficient evidence to suggest the diagnosis of HCM. In this case, a two-dimensional approach displayed limitations in capturing septal hypertrophy on multiple occasions. In addition, this case demonstrated an increased sensitivity and specificity of a three-dimensional approach, such as that provided by cardiac MRI. This report highlights the need for considering HCM in patients with DOE, chest pain, and dizziness between the ages of 40 and 70 and the necessity of cardiac MRI in patients with repeated occurrences of symptoms when TTE imaging does not reveal significant abnormalities.
